# Multigene phylogeny of root-knot nematodes and molecular characterization of *Meloidogyne nataliei* Golden, Rose & Bird, 1981 (Nematoda: Tylenchida)

**DOI:** 10.1038/s41598-019-48195-0

**Published:** 2019-08-13

**Authors:** Sergio Álvarez-Ortega, Janete A. Brito, Sergei. A. Subbotin

**Affiliations:** 1 Departamento de Biología y Geología, Física y Química Inorgánica, Universidad Rey Juan Carlos, Campus de Móstoles, 28933 Madrid, Spain; 2 Division of Plant Industry, Florida Department of Agriculture and Consumer Services, Gainesville, FL 32614-7100 USA; 3 Plant Pest Diagnostic Center, California Department of Food and Agriculture, Sacramento, CA 95832-1448 USA; 4 Center of Parasitology of A.N. Severtsov Institute of Ecology and Evolution of the Russian Academy of Sciences, Leninskii Prospect 33, Moscow, 117071 Russia

**Keywords:** Taxonomy, Phylogenetics

## Abstract

The root-knot nematodes of the genus *Meloidogyne* are highly adapted, obligate plant parasites, consisting of nearly one hundred valid species, and are considered the most economically important group of plant-parasitic nematodes. Six *Meloidogyne* species: *M*. *arenaria*, *M*. *hapla*, *M*. *incognita*, *M*. *microtyla*, *M*. *naasi* and *M*. *nataliei* were previously reported in Michigan, USA. For this study, *Meloidogyne nataliei* was isolated from the grapevine *Vitis labrusca* from the type locality in Michigan, USA, and was characterized using isozyme analysis and ribosomal and mitochondrial gene sequences. No malate dehydrogenase activity was detected using macerate of one, five, six, seven or ten females of *M*. *nataliei* per well. However, one strong band (EST = S1; Rm: 27.4) of esterase activity was detected when using homogenates of ten egg-laying females per well. Phylogenetic analyses of sequences of the partial 18S ribosomal RNA, D2-D3 of 28S rRNA, internal transcribed spacer of rRNA, mitochondrial *cytochrome oxidase subunit I* genes and the *cytochrome oxidase subunit II*-16S rRNA intergeneric fragment from fifty-five valid *Meloidogyne* species and *M*. *nataliei* were conducted using Bayesian inference and maximum likelihood methods. From these results, we infer 11 distinct clades among studied species, with *M*. *nataliei* and *M*. *indica* composing a basal lineage. Seventy five percent of these species belong to seven clades within the *Meloidogyne* superclade. Characterization of these clades is provided and evolutionary trends within the root-knot nematodes are discussed.

## Introduction

The root-knot nematodes (RKNs) of the genus *Meloidogyne* are highly adapted obligate plant parasites. Their worldwide distribution and broad host range (parasitizing nearly every species of higher plant) contribute to their status as the most economically important group of plant-parasitic nematodes^1^. Nearly one hundred valid species are in the genus^2,3^, most of which are prevalent in warm temperate and tropical regions. Only six *Meloidogyne* species, namely *M*. *arenaria*, *M*. *hapla*, *M*. *incognita*, *M*. *microtyla*, *M*. *naasi* and *M*. *nataliei*, are reported in Michigan, USA^4^. The globally-distributed northern root-knot nematode, *M*. *hapla*, is the most common species in Michigan; however, it is also home to the Michigan grape root-knot nematode, *M*. *nataliei*, which was found only in vineyards, sometimes in association with *M*. *hapla*. *Meloidogyne nataliei* is characterized by its unique morphology, cytogenetics and biology. It was detected in 1977 from root samples of grape (*Vitis labrusca* cv. Concord) from a declining vineyard in Mattawan, Van Buren County, Michigan^5^. In 1980, the Michigan grape root-knot nematode became a state-mandated regulatory species for eradication. During 1983–1984 an attempt to eradicate this nematode species in the type locality was made^6^, but it was rediscovered on the original farm in 2012 and 2017^4^. While this species is morphologically and cytologically well characterized, no molecular and isozyme data are available for *M*. *nataliei*.

The wine grape and wine industry are important in Michigan, contributing to several economic sectors including jobs, retail and tourism. Currently, there are around 3,050 wine grape vineyard acres in Michigan and more than 130 producers of wine. Grapevines (*Vitis* spp.) are hosts to many species of plant-parasitic nematodes^7^ among which root-knot nematodes are the most important. They feed on or inside the roots, and symptoms often are not visible due to their feeding does not result on the production of characteristic secondary symptoms. Proper identification of root-knot nematodes is necessary in order to design environmentally friendly management strategies to control these pests.

During a nematology survey conducted at multiple sites in Southwest Michigan in 2017, several grape root and soil samples were collected from the type locality of *M*. *nataliei*. The study of nematode specimens extracted from these samples revealed the presence of the Michigan grape root-knot nematode. Here, we leverage these collected specimens to *i*) perform molecular and isozyme analysis of *M*. *nataliei; ii*) elucidate evolutionary relationships of *M*. *nataliei* with its congeners while *iii*) gaining insight on the phylogeny of the root-knot nematodes using a multigene dataset containing species reference sequences of partial 18S rRNA, the D2-D3 of 28S rRNA, ITS1 rRNA partial *COI* mtDNA gene and the *COII*-16S rRNA intergeneric fragment.

## Results

### Bionomics

**Figure 1:**
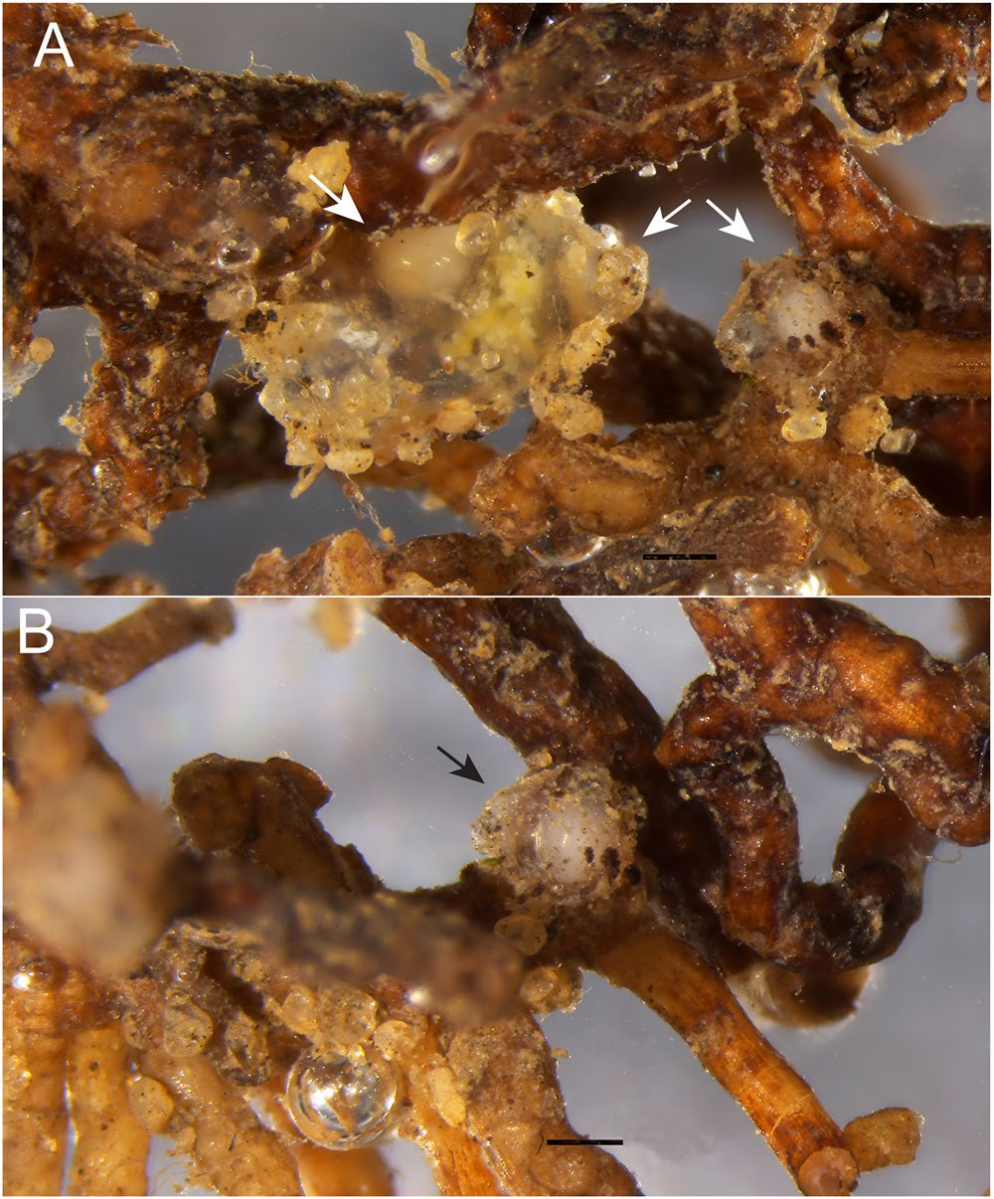
(**A,B**) Infected roots of grape with females protruding (arrows), some cover by a massive eggs mass. (Scale bars: 0.5 mm).

Grape roots infected with *M*. *nataliei* did not show galls or knots (Fig. 1). As previously mentioned in the original species description^5^, *M*. *nataliei* is an unusual root-knot nematode in that it does not form galls or knots, and only the anterior region of the female (i.e. lip region and neck) is embedded into the roots. The females of *M*. *nataliei* protrude from the root surface and they are usually cover by a massive eggs mass (Fig. 1).

### Morphological analysis

**Figure 2:**
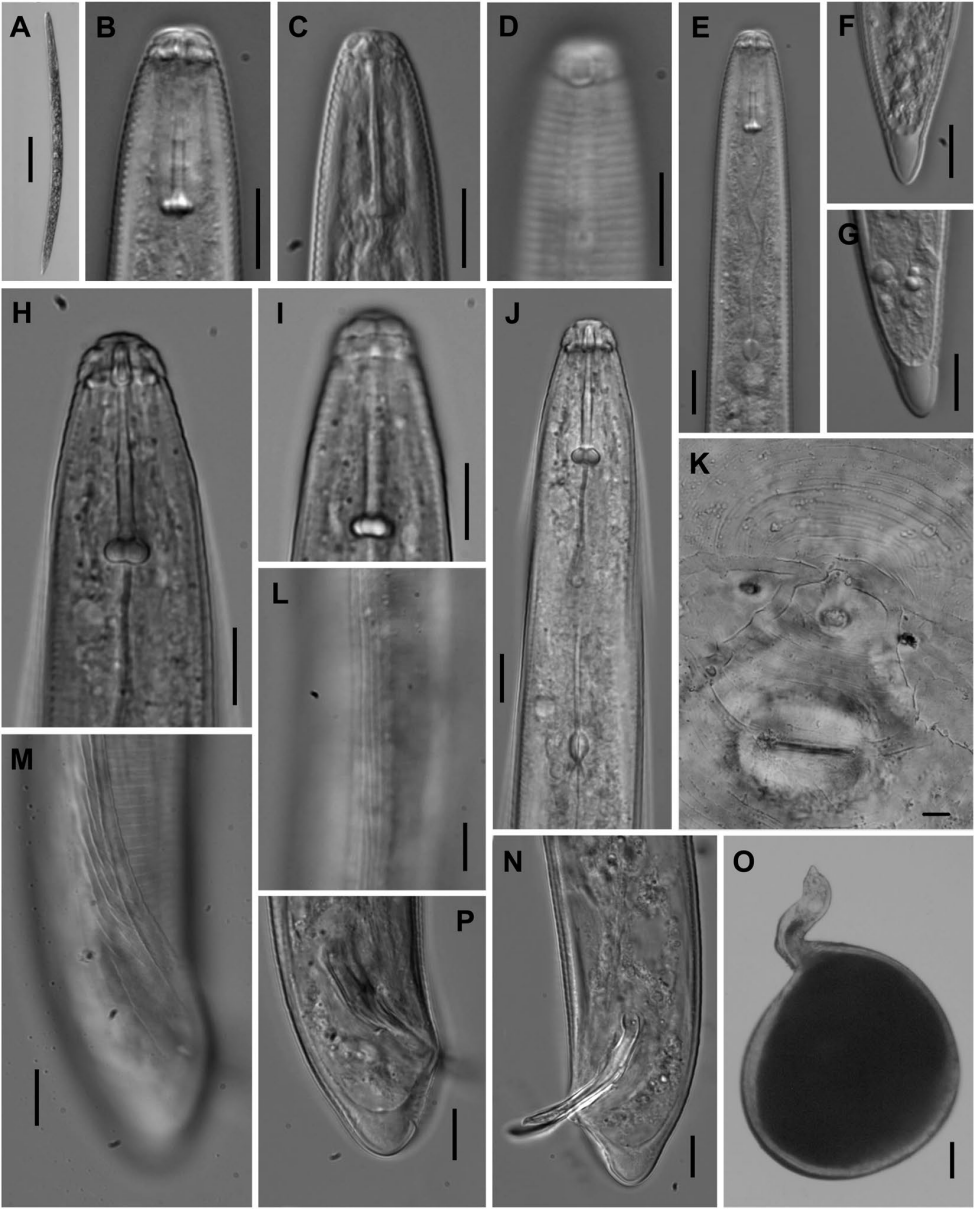
General morphology of *Meloidogyne nataliei* (LM). (**A–G**) Second-stage juveniles; (**H–J,L–N,P**): Male; (**K,O**): Female. (**A,O**) Entire body. (**B,C,H**) Anterior region in median lateral view. (**D,I**) Anterior region in surface lateral view. (**E,J**) Pharyngeal region. (**K**) Perineal pattern. (**L**) Mid-body lateral field. (**F,G,M,N,P**) Caudal region. (Scale bars: A,O: 100 µm; B–J, K–N, P = 10 µm).

Females, males and second-stage juveniles (J2s) were examined under light microscopy (Fig. 2). The general morphology of specimens perfectly fits with the original description of *M*. *nataliei*. Especially remarkable are: (i) the lip region morphology of the J2s (Fig. 2B,E) and males (Fig. 2H,J), with a heavy cephalic sclerotization, (ii) the female perineal pattern (Fig. 2K), and (iii) the tail shape the of the J2s (Fig. 2F,G), with a short hyaline portion and narrowly rounded tail terminus.

### Isozyme analysis

**Figure 3:**
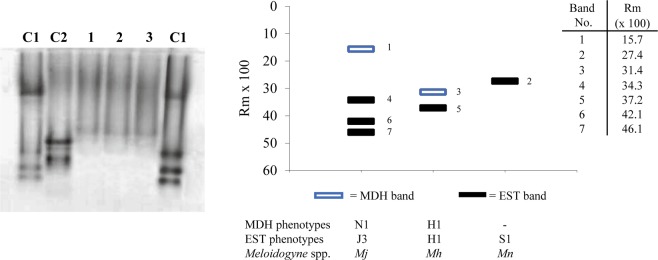
Esterase (EST) and malate dehydrogenase (MDH) phenotypes from egg-laying females of *Meloidogyne nataliei*, and *M*. *javanica* and *M*. *hapla* used as controls. C1- extract from one female of *M*. *javanica* (J3; N1) per well; C2 - extract of five females of *M*. *hapla* (H1; H1) per well; Lanes 1, 2, and 3 - extract from five females of *M*. *nataliei* (S1) per well.

No clear resolution was detected for EST activity when using extracts of five egg-laying females of *M*. *nataliei* per well (Fig. 3). However, two bands, one weak (minor) and one strong (phenotype S1) of EST activity were clearly detected when using homogenates of more than five egg-laying females (six or ten) per well (Supplement Fig. S1). No MDH activity was detected regardless of the number of *M*. *nataliei* female extracts added per well (Fig. 3). The *M*. *hapla* (H1; H1) and *M*. *javanica* (J3; N1) controls yielded the species-specific EST and MDH phenotypes. The EST pattern for *M*. *nataliei* was clearly different from the EST pattern resolved for *M*. *hapla* and *M*. *javanica*.

### Molecular analysis

**Figure 4:**
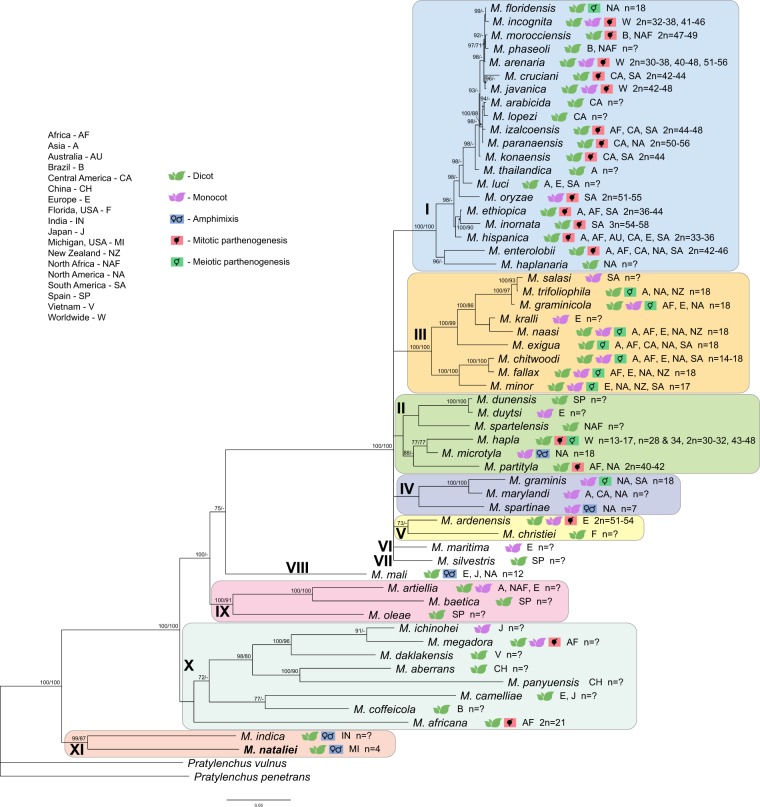
Bayesian 50% majority rule consensus tree as inferred from 18S rRNA, ITS1 rRNA, D2-D3 expansion segments of 28S rRNA, *COI* gene and *COII*-16S rRNA sequence alignment under the GTR + I + G model. Branch support of over 70% is given for appropriate clades and it is indicated as: posterior probabilities value in Bayesian inference analysis/bootstrap value from maximum-likelihood analysis. (n = ? – chromosome number information unknown).

Reference sequences of fifty-five valid *Meloidogyne* species available from the GenBank database and newly obtained sequences of *M*. *nataliei* were used for phylogenetic analysis (Supplement Table S1). The molecular phylogenetic trees obtained for the five genes are presented in Supplement Figs S2–S5, S7, S8 and Fig. 4.

Amplification of partial 18S rRNA gene yielded a single 834 bp fragment. A BLAST search of *M*. *nataliei* 18S rRNA gene sequence revealed the highest matches – 93.1–93.3% (99% coverage) – with the sequences of *Zygotylenchus* spp., *Hirschmanniella* spp., *Trophurus* spp., *Bitylenchus* spp. and *Tylenchorhynchus* spp. The phylogenetic tree reconstructed from the 18S rRNA gene sequence analysis (44 *Meloidogyne* species, 1709 bp alignment length) (Fig. S2) showed the highly supported superior clade (PP and BS = 100%) with four clades (*M*. *javanica-M*. *enterolobii; M*. *hapla-M*. *silvestris; M*. *chitwoodi-M*. *exigua; M*. *christiei*). Other nematodes were distributed among six clades (*M*. *mali; M*. *baetica-M*. *oleae; M*. *camelliae-M*. *aberrans; M*. *coffeicola; M*. *africana; M*. *nataliei*). *Meloidogyne nataliei* occupied a basal position within the genus.

Amplification of the D2-D3 expansion segments of 28S rRNA gene yielded a single 716 bp fragment. A BLAST search of *M*. *nataliei* D2-D3 of 28S rRNA gene sequence revealed the highest match with the sequence of *Meloidogyne indica* (MF680038), the identity of *M*. *nataliei* D2-D3 of 28S rRNA gene sequence with that of *M*. *indica* was only 86% (99% coverage). The phylogenetic tree obtained (Fig. S3) from the D2-D3 of 28S rRNA gene sequence analysis (42 *Meloidogyne* species, 791 bp) showed a highly-supported superior clade (PP and BS = 100%), which included the four major *Meloidogyne* clades (*M*. *arabicida-M*. *inornata; M*. *spartelensis-M*. *hapla; M*. *trifoliophila-M*. *minor; M*. *graminis* with *M*. *maylandi*) and the clade with *M*. *christiei*. Other nematodes were distributed among six clades (*M*. *mali; M*. *artiellia-M*. *oleae; M*. *camelliae; M*. *daklakensis-M*. *aberrans; M*. *africana; M*. *indica* with *M*. *nataliei*). *Meloidogyne nataliei* had a sister relation with *M*. *indica* and occupied a basal position within the genus.

Amplification of ITS rRNA gene yielded a single 696 bp fragment. A BLAST search of *M*. *nataliei* ITS rRNA region sequence revealed the highest match with the ITS sequence of *Hirschmanniella* spp., *Pratylenchus* spp. and *Tylenchorhynchus clarus*, the identity of *M*. *nataliei* ITS rRNA gene sequence with these sequences were 82–84%, but with only 51–55% coverage. Because of high numbers of ambiguity in the ITS2 region alignment, only the alignment fragment containing the ITS1 and partial 5.8S rRNA gene was used in the phylogenetic analysis. The tree obtained from the full length ITS1 alignment (40 *Meloidogyne* species, 522 bp) is given in Fig. S4. Since, the ITS1 alignment also showed some ambiguous regions, Gblocks was used to delete the divergent sections to create the culled ITS1 alignment, which was 325 bp in a length. The phylogenetic positions of most of *Meloidogyne* spp. reconstructed from the culled ITS1 alignment (Fig. S5) were generally congruent with those obtained from the full length ITS1 alignment. *Meloidogyne nataliei* formed a clade with *M*. *indica* and occupied a basal position within the genus in the tree inferred from the culled ITS1 alignment.

A total of 258 sequences of 13 valid and an unidentified species belonging to the root-knot tropical species group was used for the analysis. The alignment was 454 bp in a length. A phylogenetic network with the ITS rRNA sequences reconstructed using SP with POPART software is given in Fig. S6. This method revealed two species groups (*Incognita* and *Ethiopica*) and *M*. *enterolobii*. Maximal sequence variation for the clade 1 was 15.5%, for the *Incognita* group – 11%, for the *Ethiopica* group – 3.5% and for *M*. *enterolobii* – 10.1%. The ITS diversity was structured into two groups for *M*. *enterolobii*, *M*. *paranaensis* and some species of the *Ethiopica* group, which ITS paralogs clustered with the *Incognita* group.

Amplification of the partial *COI* gene yielded a single 439 bp fragment. A BLAST search of *M*. *nataliei COI* sequence revealed the highest match (83.1% with 100% coverage) with the *COI* sequence of *M*. *camelliae* (KM887147). The phylogenetic tree inferred from the *COI* gene analysis (31 *Meloidogyne* spp., 433 bp) is given in Fig. S7. Phylogenetic relationships between most clades and species were poorly resolved. *Meloidogyne nataliei* formed a basal lineage within the genus.

The 567-bp *COII*-16S rRNA intergeneric fragment alignment included 31 sequences of root-knot nematodes. The phylogenetic tree inferred from this fragment is given in Fig. S8.

The combined alignment consists of five genes from 56 *Meloidogyne* species and was 4022 bp in length. The inferred phylogenetic tree contained eleven clades (Fig. 4). *M*. *nataliei* and *M indica* formed a basal clade to all other *Meloidogyne* species. Mapping of some biological and biogeographical characters: (*i*) host range; (*ii*) mode of reproduction; (*iii*) chromosome number, and *iv*) species distribution are also given in Fig. 4.

## Discussion

### Isozyme analysis

The isozymes resolved from egg-laying females of *M*. *nataliei* revealed a single major band (EST = S1 phenotype) and a single weak band of esterase activity^8^. Poorly defined weak isozyme band patterns have been reported for several *Meloidogyne* spp. including *Meloidogyne haplanaria*^9^, *M*. *dunensis*^10^, and *M*. *silvestris*^11^. These weak bands are considered minor bands and believed to have low value for identification of *Meloidogyne* spp. They are inconsistent and often depend on increasing the quantity of females homogenized added per well^8^. Although the EST S1 pattern resolved for *M*. *nataliei* is similar for that reported for *M*. *chitwoodi* (MDH = N1a) and *M*. *platani* (MDH = N1a), *M*. *nataliei* clearly differed from these RKN species because no MDH activity could be detected.

### Phylogenetic relationships within the genus *Meloidogyne*

Tandingan De Ley *et al*.^12^ were the first who used 18S rRNA gene sequences for a rigorous reconstruction of *Meloidogyne* phylogeny. This analysis, which included only 12 species of *Meloidogyne* and four outgroup taxa, revealed three clades (I, II and III) within the genus. Later, Tigano *et al*.^13^, Holterman *et al*.^14^, Kiewnick *et al*.^15^ and Janssen *et al*.^16^ using same gene and but larger number of species confirmed the presence of these three major clades within *Meloidogyne*. Several other genes were also successfully used for reconstruction of *Meloidogyne* single gene phylogeny: the ITS rRNA^3,11,17–24^, the D2-D3 of 28S rRNA^3,11,15,18–25^; the RNA polymerase II gene (*rpb1*)^26,27^, the region between the *COII* and 16S rRNA genes of mtDNA^3,20,22–24,28^; *hsp90*^29^, *COI*^3,15,16,24,30,31^ and IGS rRNA^32^, *COII*^15^. However, it has been shown by Hugall *et al*.^33^ and confirmed by the present study that the ITS rRNA gene of species from the tropical group can contain highly polymorphic copies, which may disrupt phylogenetic reconstruction. Several authors reconstructed the multigene *Meloidogyne* phylogeny using supertree^34^ or supermatrix^16,35^ approaches resulting in more resolution of relationships.

In our study we estimated a multigene tree from the dataset containing reference sequences of five genes of 56 *Meloidogyne* species, which represents more than half of known valid species of root-knot nematodes. Our phylogenetic analysis and several published phylogenies of *Meloidogyne* revealed that not all species can be assigned to the three major clades. In this study we proposed a new clade numbering as an attempt to classify all studied species. The root-knot nematode species studied here are distributed among eleven highly or moderately supported clades, seven of which compose a “superclade,” containing 75% of the studied species. The superclade contains the clade I and the clade III *sensu* Tandingan De Ley *et al*.^12^. Species from the former clade II are designated into new clades II, IV, VI and VII.

The evolutionary trends of the root-knot nematodes that compose the superclade have been characterized by Tandingan De Ley *et al*.^12^, Tigano *et al*.^13^ and Holterman *et al*.^14^. Here, through the incorporation of additional taxa, we are able to characterize these observed patterns more robustly.

Clade I includes *Meloidogyne* species distributed in warmer climates and contains *Meloidogyne incognita*, *M*. *javanica*, *M*. *arenaria* and 17 other species which are commonly referred as the tropical root-knot nematode complex. Three species of this complex: *M*. *arenaria*, *M*. *incognita* and *M*. *javanica* belonging to Incognita group are globally distributed, polyphagous pests of many agricultural crops. *Meloidogyne phaseoli* and *M*. *morocciensis* are found in Brazil and North Africa, whereas *M*. *floridensis* is known only from Florida and California, USA. *Meloidogyne arabicida*, *M*. *izalcoensis*, *M*. *lopezi*, *M*. *paranensis* and *M*. *konaensis* parasitize coffee tree and other dicots in North, Central, and South America. *Meloidogyne thailandica*, and the representatives of Ethiopica group^36^ (*M*. *luci*. *M*. *ethiopica*, *M*. *inornata* and *M*. *hispanica*) infect different dicots and have been reported from Asia, Africa, South America and Europe.

The mitotic parthenogenetic *Meloidogyne oryzae* was considered by several authors in clade III^13,14^. However, the results obtained by Negretti *et al*.^37^ and da Silva Mattos *et al*.^38^ showed that the sample used for 18S rRNA gene sequencing by Tigano *et al*.^13^ was mistakenly identified and it indeed belonged to *M*. *graminicola*. The present results with the ITS rRNA gene sequence deposited by da Silva Mattos *et al*.^38^ showed that *M*. *oryzae* belongs to clade I. Accepting da Silva Mattos *et al*.*’s*^38^ identification of *M*. *oryzae* and ITS rRNA gene sequence (KY962653-isolate Mo1, KY962654-isolate Mo2), we consider the sequences of the 18S rRNA (AY942631) by Tigano *et al*.^13^, *COI* (MH128473, MH128474) by Powers *et al*.^31^ and the D2-D3 of 28S rRNA (KY962662-isolate Mo2) by da Silva Mattos *et al*. (unpublished) as belonging to *M*. *graminicola* or other unknown species.

It has been shown by nuclear genome sequencing that *M*. *incognita*, *M*. *javanica* and *M*. *arenaria* contain divergent copies of many loci. The different evolutionary histories of these copies, likely arising via historical hybridization and genome duplications, complicate both phylogenetic analyses and species identifications that rely on nuclear gene sequences^26,33,39^. Diagnosis of the majority of *Meloidogyne* species is difficult, and is primarily based on mtDNA genes, namely *ND5* and the region between *COII* and 16S, or isozymes. All species of this clade for which reproductive mode is known are polyploids and exclusively comprise mitotic parthenogenetic species, except for the meiotic parthenogenetic *M*. *floridensis*.

Clade II contains six species that parasitize monocots and dicots. It includes *M*. *microtyla* from North America and *M*. *hapla*, which is a major pest of many crops worldwide. The first species have different modes of reproduction: mitotic parthenogenesis and amphimixis, whereas *M*. *hapla* has two reproduction modes: meiotic and mitotic parthenogenesis depending on race. *Meloidogyne partityla*, from Africa and North America, reproduces by mitotic parthenogenesis. And *M*. *spartelensis*, *M*. *duytsi*, and *M*. *dunensis* have unknown reproductive strategies and are presently found in Europe or North Africa.

Clade III contains nine species parasitizing monocots and dicots and is primarily distributed in several continents, except for *M*. *salasi* found in South America and *M*. *kralli* from Europe. Species from this group are exclusively meiotic parthenogenetic.

The clade IV contains *M*. *spartinae*, *M*. *marylandi* and *M*. *graminis* and its host range seems to be limited to the Poaceae only. These species have likely North American origin, although *M*. *graminis* was reported from Europe and South America and *M*. *marylandi* is also found in Asia. *Meloidogyne spartinae* is amphimictic species, whereas *M*. *graminis* is exclusively meiotic parthenogenetic one.

The clade V includes *M*. *christiei*, that has been found to only parasitize Turkey oak roots, and *M*. *ardenensis*. *Meloidogyne christiei* forms small galls involving the tissues immediately surrounding the nematode. Although mode of reproduction for this species is unknown, the presence of many males in populations may indicate amphimixis.

Both Clades VI and VII are monotypic; VI contains *M*. *maritima* that infects *Ammophila arenaria* on dunes at Perranporth, Cornwall, UK, while VII is represented by *M*. *silvestris* which parasitizes the roots of European holly, *Ilex aquifolium*, in Soria province, Spain.

The clade VIII includes only the amphimictic species *M*. *mali* which parasitizes trees and woody plants of the genera *Ulmus*, *Euonymus*, *Acer* and others in Japan. This species was likely introduced to North America and Europe from Asia.

Clade IX includes the species *M*. *artiellia*, *M*. *baetica* and *M*. *oleae*. The first species was found in Europe, Asia and North Africa, parasitizing monocots and dicots, whereas the two other species are reported in Spain from olive trees and other plants. Mode of reproduction and chromosome number for this group are still unknown.

Clade X includes eight species: *M*. *aberrans*, *M*. *panyuensis*, *M*. *camelliae*, *M*. *ichinohei*, *M*. *daklakensis*, from East Asian, *M*. *megadora* and *M*. *africana* from Africa and *M*. *coffeicola*, from Brazil, which parasitizes coffee trees. These species parasitize monocots and dicots. The reproductive mode is only known for two representatives of this clade: *M*. *megadora* and *M*. *africana*; both exhibit mitotic parthenogenesis. *Meloidogyne africana* has the lowest number of chromosomes (2n = 21) of RKNs known to reproduce by mitotic parthenogenesis^16^.

Finally, clade XI represents the earliest branching lineage of RKNs and includes two species, *M*. *indica* and *M*. *nataliei*.

Based on analysis of many morphological characters, Jepson^40^ proposed 12 morphological groups within J2s, six groups of perineal patterns within females, and seven groups based on head morphology in males. She placed all studied species in several *Meloidogyne* groups named according to the oldest described species within them, namely, “*graminis*”, “*acroneae*”, “*exigua*”, “*nataliei*” and others. Although none of these groupings fit exactly with the groups proposed in the present study, there are some patterns of overlap, which require careful analysis and whose characterization would benefit from inclusion of more species.

### Evolutionary relationships of *Meloidogyne nataliei* with other nematodes

The phylogenies obtained for the four loci analyzed for *M*. *nataliei* revealed sufficient divergence of this root-knot nematode species to demonstrate its uniqueness among the other species of *Meloidogyne*. Our failure to detect MDH via isozyme analysis supports previous results for *M*. *nataliei*; this apparent lack is an unusual trait among *Meloidogyne* spp.^41^.

The phylogenetic trees obtained from the D2-D3 of 28S and ITS rRNA gene datasets and the multigene tree showed that the Michigan grape root-knot nematode clustered with *M*. *indica*, in a highly-supported clade. Both species are morphologically similar. Jepson^40^ distinguished 12 morphological groups within J2s of the genus *Meloidogyne* and placed *M*. *nataliei*. *M*. *indica*, *M*. *brevicauda* and *M*. *propora* in the group 1. This group is characterized by a tapering tail terminus with a very broad and rounded tip in J2s. Jepson^40^ noted small qualitative differences between species within the group, but they were not easily defined in practice. *Meloidogyne indica* and *M*. *brevicauda* were reported from the Indian subcontinent, and *M*. *propora* was found in the Outer Islands of the Seychelles. While the three abovementioned species are tropically distributed, *M*. *nataliei* is from Michigan, which exhibits a humid continental climate, and is among the coldest regions in the contiguous United States. We hypothesize that *M*. *nataliei* is an invasive species for Michigan, which was likely introduced with its plant-host, *Vitis labrusca*, one of 17 species native to the warmer climate Southeastern USA^42^. If this is the case, future nematological surveys in this region will likely broaden the known distribution of *M*. *nataliei*.

Our results suggest that *M*. *nataliei* together with *M*. *indica* represents an early branching lineage of root-knot nematodes, which exhibit shared, perhaps ancestral characteristics. Molecular results reject a hypothesis by Goldstein and Triantaphyllou^43^, who suggested that the Michigan grape root-knot nematode might not belong to *Meloidogyne*. Phani *et al*.^44^ provided a molecular characterization of *M*. *indica* and already noticed that this root-knot nematode species should be considered the most ancestral taxon of the genus, based on molecular data.

*Meloidogyne nataliei* is a diploid amphimictic species that has a haploid complement of only four chromosomes^41^. The four chromosomes of *M*. *nataliei* are relatively large when compared to those of other *Meloidogyne* species. Moreover, *M*. *nataliei* reproduces exclusively by cross-fertilization. Our results support the hypothesis of Triantaphyllou^45,46^ that amphimictic *Meloidogyne* species with low chromosome count are ancestral traits, from which the mitotic parthenogenetic RKN species have evolved.

Ryss^47^ considered pratylenchids as the most closely related to *Meloidogyne* due to the shared morphological characteristics of the lip region and pharyngeal structure, and these morphological similarities might be indicative of common ancestry between Meloidogynidae and Pratylenchidae. Close relationship of *Meloidogyne* with Pratylenchidae is further supported by phylogenetic analysis of the D2-D3 region of 28S rRNA^48^. Using 18S rRNA gene to estimate the tylenchid phylogeny, Holterman *et al*.^14^ also suggested that that root-knot nematodes have evolved from a *Pratylenchus*-like ancestor. Surprisingly, the morphology of the lip region of *M*. *nataliei* J2 juveniles and males is heavily sclerotized, resembling the pattern observed in *Pratylenchus*. A strong stylet and tail with widely rounded terminus in *M*. *nataliei* J2 juveniles are resemble those of some *Pratylenchus*. These features may well represent the plesiomorphic condition of the genus *Meloidogyne*.

### Evolutionary trends within the root-knot nematodes

Triantaphyllou^45^ summarized the cytogenetics of root-knot nematodes and suggested that obligate amphimictic species with n = 18 or 19 should be considered as closely related to the ancestors of *Meloidogyne* spp. He also believed that the low chromosomal numbers in most other nematodes offered support for a polyploid origin of most *Meloidogyne* species. Janssen *et al*.^16^ concluded that that the basic haploid chromosome number of the genus *Meloidogyne* could possibly be as low as n = 7. The phylogenetic placement of the amphimictic *M*. *nataliei* with n = 4 at a basal position to all other *Meloidogyne* species supports the hypothesis of a low chromosome number in ancestral species.

Castagnone-Sereno *et al*.^49^ noticed that the extensive diversity within RKNs in terms of chromosome complement is the reflects a complex evolution within the genus, involving genome duplication, polyploidization, introgression and hybridization. The shift in reproductive mode, along with the evolution of broad polyphagy likely coincides with the rapid diversification of the *Meloidogyne* superclade, which might explain unresolved relationships between clades I-VII. The occurrence of parthenogenesis has correlated in root-knot nematodes with its increasing importance as crop parasites^14^.

It has been suggested that the current apomictic species derived from diploid sexual ancestors while obligatory parthenogenetic mitotic species evolved from facultatively parthenogenetic meiotic species, following suppression of meiosis during oocyte maturation^50^. However, the mapping of reproductive mode on a multigene phylogenetic estimate^16^ along with the results presented here do not support this hypothesis, rather suggesting that the transition to mitotic parthenogenesis may have occurred earlier than the appearance of meiotic parthenogenesis in root-knot nematode evolution.

## Material and Methods

### Nematode population

Soil and grape root samples were collected in a vineyard (*Vitis labrusca*), with clear symptoms of decline, in Mattawan, Michigan (42°12′16.1″N, 85°46′53.3″W; 42°12′15.4″N, 85°46′53.1″W; 42°12′16.7″N, 85°46′53.3″W). Nematodes were extracted from soil samples by sieving and sucrose centrifugation technique, somewhat modified (density = 1.18) from Barker^51^.

### Morphological study

Second-stage juveniles, males and females were manually picked from nematode suspension and root-galls under a binocular microscope using a dissecting needle. Several specimens were mounted on temporary glass slides for observation under light microscopy (LM). Some of the best-preserved specimens were photographed with a Nikon Eclipse 80i and Olympus BX51 microscope equipped with DIC and digital cameras.

### Isozyme analysis

Frozen egg-laying females (8 to 10 day-old) previously dissected directly from grape root were subjected to isozyme analysis, namely esterase (EST) and malate dehydrogenase (MDH). Each female was placed in a 0.6-ml microfuge tube containing 5 µl of deionized water and an equal volume of sample buffer (BioRad, Hercules, CA). Individual females were macerated and 10 µl of extract was transferred onto wells of a polyacrylamide gel consisting of a 4% stacking (pH 6.8) and 8% separating gel (pH 8.8). The gel was placed in Tris-glycine buffer (pH 8.3) contained in a Mini PROTEAN III unit (BioRad). Extracts from *M*. *hapla* and/or *M*. *javanica* females were loaded separately to individual wells on each gel as a comparative reference for *M*. *nataliei*. Because no MDH activity was detected when using extract from a single female of *M*. *nataliei*, the number of specimens per well was increased to five, six, seven and ten females. A total of 80 females were examined. Electrophoresis was carried as previously reported^52^ and detection of bands carried out by staining gels for EST with 100 ml of substrate solution (3 ml α-naphthyl acetate [1% in 50% acetone], 100 mg Fast Blue RR Salt, and 0.05 M Potassium phosphate buffer, pH 6.0) and for MDH with 100 ml of the staining solution (0.02 g thiazolyl blue tetrazolium bromide, 0.026 g β-nicotinamida adenine dinucleotide, 0.076 g L (-) malic acid, 0.006 g phenazine methosulfate, and 0.05 M Tris-HCL, pH 8.6) (Sigma – Aldrich, St. Louis, MO)^8^ for 45 min and 15 min respectively, at 37 °C in the dark. Relative migration of major bands was calculated, and phenotype designations were assigned^53^.

### DNA extraction, PCR and sequencing

DNA was extracted from several juveniles using the proteinase K protocol. Crushed specimens were transferred to an Eppendorf tube containing 16 μl double distilled water, 2 μl 10X PCR buffer and 2 μl proteinase K (600 *μ*g/ml) (Promega, Madison, WI, USA). The tubes were incubated at 65 °C (1 h) and then at 95 °C (15 min). Detailed protocols for PCR, cloning and sequencing were as described by Tanha Maafi *et al*.^54^. Three rRNA gene fragments (ITS rRNA, D2-D3 expansion segments of 28S rRNA; partly 18S rRNA) and partial *COI* mtDNA gene were amplified and used for phylogenetic analysis. The following primers were used for amplification in the present study: ITS-rRNA – TW81 (5′-GTT TCC GTA GGT GAA CCT GC-3′) and AB28 (5′-ATA TGC TTA AGT TCA GCG GGT-3′)^55^; D2-D3 of 28S rRNA – D2A (5′-ACA AGT ACC GTG AGG GAA AGT TG -3′) and D3B (5′-TCG GAA GGA ACC AGC TAC TA-3′)^48^; 18S rRNA – G18SU (5′-GCT TGT CTC AAA GAT TAA GCC-3′) and R18Tyl1 (5′-GGT CCA AGA ATT TCA CCT CTC-3′)^56^, *COI* – JB3 (5′-TTT TTT GGG CAT CCT GAG GTT TAT-3′) and JB5 (5′-AGC ACC TAA ACT TAA AAC ATA ATG AAA ATG-3′)^57^. PCR products were purified using the QIAquick PCR purification Kit (Qiagen, Hilden, Germany) and used for direct sequencing. The newly obtained sequences have been submitted to the GenBank database under the numbers: MG821326-MG821329 for D2-D3 of 28S rRNA, 18S rRNA, ITS rRNA and *COI* genes, respectively.

### Phylogenetic study

The new sequences of 18S rRNA, D2-D3 of 28S rRNA, ITS rRNA, and *COI* genes of *M*. *nataliei* were aligned using ClustalX 1.83 with their corresponding published gene sequences of *Meloidogyne* species^11–14,16,18,22–25,31,35,58^ (Table S1). Two alignments were created for the ITS rRNA gene sequences: (i) full length alignment and (ii) culled alignment. For the culled alignment, poorly aligned and divergent regions were eliminated using the online version of Gblocks 0.91b^59^ under the option “Allow gap positions within the final blocks” for less stringent parameters (http://molevol.cmima.csic.es/castresana/Gblocks_server.html). The alignment of the *COII*-16S rRNA intergeneric region^35^ with additional species^23^ was also included in the study. Outgroup taxa for each data set were chosen according to the results of previously published data^14,15,18^. Alignments for each gene fragment and combined alignment containing all genes were separately analyzed with Bayesian inference (BI) and maximum likelihood (ML). BI and ML analyses of the sequence dataset were performed at the CIPRES Science Gateway^60^, using MrBayes 3.2.6^61^ and RAxML 8.2.10^62^, respectively. The best fit model of DNA evolution for each gene fragment was estimated under Akaike Information Criterion (AIC) using jModelTest 2.1.10^63^. BI analysis was initiated with a random starting tree and run with the four Metropolis-coupled Markov chain Monte Carlo (MCMC) for 2 × 10^6^ generations. The Markov chains were sampled at intervals of 100 generations. Two runs were performed for each analysis. After discarding burn-in samples and evaluating convergence, the remaining samples were retained for further analysis. ML analysis was implemented under the same nucleotide substitution model as in the BI, and 1000 bootstrap replications. The topologies were used to generate a 50% majority rule consensus tree. Bayesian posterior probabilities (PP) and ML bootstrap support (BS) of over 70% are given on appropriate clades.

The ITS rRNA gene sequences of species from the clade I were downloaded from the GenBank and aligned using ClustalX 1.83. The alignment for ITS rRNA was used to construct phylogenetic network estimation using statistical parsimony (SP) as implemented in POPART software (http://popart.otago.ac.nz)^64^.

The trees and network were visualised with the program TreeView 1.6.6 and FigTree v1.4.3 and drawn with Adobe Illustrator CC.

## Supplementary information

Supplementary information

## References

[1] Moens, M., Perry, R. N. & Starr, J. L. *Meloidogyne* species – a diverse group of novel and important plant parasites in *Root-knot Nematodes* (eds Perry, R. N., Moens, M. & Starr, J. L.) 1–17 (CAB International: Cambridge, MA, 2009).

[2] Hunt, D. J. & Handoo, Z. A. Taxonomy, identification and principal species in *Root-knot Nematodes* (eds Perry, R. N., Moens, M. & Starr, J. L.) 55–97 (CAB International: Cambridge, MA, 2009).

[3] Trinh QP (2019). *Meloidogyne daklakensis* n. sp. (Nematoda: Meloidogynidae), a new root-knot nematode associated with Robusta coffee (*Coffea canephora* Pierre ex A. Froehner) in the Western Highlands, Vietnam. J Helminthol.

[4] Bird, G. W. & Warner, F. Nematodes and nematologists of Michigan in *Plant Parasitic Nematodes in Sustainable Agriculture of North America*. *Vol*. 2 *– Northeastern*, *Midwestern and* Southern USA (eds Subbotin, S. A. & Chitambar, J. J.) 57–86 (Springer, 2018).

[5] Golden AM, Rose LM, Bird GW (1981). Description of *Meloidogyne nataliei* n. sp. (Nematoda: Meloidogynidae) from grape (*Vitis labrusca*) in Michigan, with SEM observations. J Nematol.

[6] Bird G, Diamond C, Warner F, Davenport J (1994). Distribution and regulation of *Meloidogyne nataliei*. J Nematol.

[7] Gutiérrez-Guttiérrez C, Palomares-Rius JE, Jiménez-Diaz RM, Castillo P (2011). Host suitability of *Vitis* rootstocks to root-knot nematodes (*Meloidogyne* spp.) and the dagger nematode *Xiphinema index*, and plant damage caused by infections. Plant Pathol.

[8] Esbenshade, P. R & Triantaphyllou, A. C. Electrophoretic methods for the study of root-knot nematodes enzymes in *An Advanced Treatise on Meloidogyne*. *Vol II*, *Methodology*. (eds Barker, K. R., Carter, C. C. & Sasser, J. N.) 115–123 (North Carolina State University, Raleigh, NC, USA, 1985).

[9] Eisenback JD, Bernard EC, Starr JL, Lee TA, Tomaszewski EK (2003). *Meloidogyne haplanaria* n. sp. (Nematoda: Meloidogynidae), a root-knot nematode parasitizing peanut in Texas. J Nematol.

[10] Palomares-Rius JE (2007). A new root-knot nematode parasitizing sea rocket from Spanish mediterranean coastal dunes: *Meloidogyne dunensis* n. sp. (Nematoda: Meloidogynidae). J Nematol.

[11] Castillo P (2009). A new root-knot nematode, *Meloidogyne silvestris*n. sp. (Nematoda: Meloidogynidae), parasitizing European holly in northern Spain. Plant Pathol.

[12] Tandingan De Ley I (2002). Phylogenetic analyses of *Meloidogyne* small subunit rDNA. J Nematol.

[13] Tigano MS, Carneiro R, Jejaprakash A, Dickson DW, Adams B (2005). Phylogeny of *Meloidogyne* spp. based on 18S rDNA and mitochondrial sequences. Nematology.

[14] Holterman M (2009). Small subunit rDNA-based phylogeny of the Tylenchida sheds light on relationships among some high-impact plant-parasitic nematodes and the evolution of plant feeding. Phytopathology.

[15] Kiewnick S (2014). Comparison of two short DNA barcoding loci (COI and COII) and two longer ribosomal DNA genes (SSU & LSU rRNA) for specimen identification among quarantine root-knot nematodes (*Meloidogyne* spp.) and their close relatives. Eur J Plant Pathol.

[16] Janssen T, Karssen G, Topalović O, Coyne D, Bert W (2017). Integrative taxonomy of root-knot nematodes reveals multiple independent origins of mitotic parthenogenesis. PLoS ONE.

[17] De Ley IT (1999). Phylogenetic analyses of internal transcribed spacer region sequences within. Meloidogyne. J Nematol.

[18] Castillo P, Vovlas N, Subbotin S, Troccoli A (2003). A New root-knot nematode, *Meloidogyne baetica* n. sp. (Nematoda: Heteroderidae), parasitizing wild olive in Southern Spain. Phytopathology.

[19] Landa BB (2008). Molecular characterization of *Meloidogyne hispanica* (Nematoda, Meloidogynidae) by phylogenetic analysis of genes within the rDNA in *Meloidogyne* spp. Plant Dis.

[20] McClure MA, Nischwitz C, Skantar AM, Schmitt ME, Subbotin SA (2012). Root-knot nematodes in golf course greens of the western United States. Plant Dis.

[21] Trisciuzzi N (2014). Detection of the camellia root-knot nematode *Meloidogyne camelliae* Golden in Japanese *Camellia bonsai* imported into Italy: integrative diagnosis, parasitic habits and molecular phylogeny. Eur J Plant Pathol.

[22] Ali N (2015). A new root-knot nematode *Meloidogyne spartelensis* n. sp. (Nematoda: Meloidogynidae) in Northern Morocco. Eur J Plant Pathol.

[23] Tao Y (2017). *Meloidogyne aberrans* sp. nov. (Nematoda: Meloidogynidae), a new root-knot nematode parasitizing kiwifruit in China. PLoS ONE.

[24] Archidona-Yuste A (2018). Diversity of root-knot nematodes of the genus *Meloidogyne* Göeldi, 1892 (Nematoda: Meloidogynidae) associated with olive plants and environmental cues regarding their distribution in southern Spain. PLoS ONE.

[25] Tenente GCMV, De Ley P, Tandingan De Ley I, Karssen G, Vanfleteren JR (2004). Sequence analysis of the D2/D3 region of the large subunit rDNA from different *Meloidogyne* isolates. Nematropica.

[26] Lunt DH (2008). Genetic tests of ancient asexuality in root knot nematodes reveal recent hybrid origins. BMC Evol Biol.

[27] Rybarczyk-Mydlowska K (2013). Both SSU rDNA and RNA polymerase II data recognise that root-knot nematodes arose from migratory Pratylenchidae, but probably not from one of the economically high-impact lesion nematodes. Nematology.

[28] Humphreys-Pereira DA, Elling AA (2014). Mitochondrial genomes of *Meloidogyne chitwoodi* and *M*. *incognita* (Nematoda: Tylenchina): Comparative analysis, gene order and phylogenetic relationships with other nematodes. Mol Biochem Parasitol.

[29] Nischwitz C (2013). Occurrence of *Meloidogyne fallax* in North America, and molecular characterization of *M*. *fallax* and *M*. *minor* from U.S. golf course greens. Plant Dis.

[30] García LE, Sánchez-Puerta MV (2015). Comparative and evolutionary analyses of *Meloidogyne* spp. Based on mitochondrial genome sequences. PloS one.

[31] Powers T, Harris T, Higgins R, Mullin P, Powers K (2018). Discovery and identification of *Meloidogyne* species using COI DNA barcoding. J Nematol.

[32] Onkendi EM, Moleleki LN (2013). Detection of *Meloidogyne enterolobii* in potatoes in South Africa and phylogenetic analysis based on intergenic region and the mitochondrial DNA sequences. Eur J Plant Pathol.

[33] Hugall A, Stanton J, Moritz C (1999). Reticulate evolution and the origins of ribosomal internal transcribed spacer diversity in apomictic *Meloidogyne*. Mol Biol Evol.

[34] Adams, B. J., Dillman, A. R. & Finlinson, C. Molecular taxonomy and phylogeny in *Root-knot nematodes* (eds Perry, R.N., Moens, M. & Starr, J. L.) 119–138 (CAB International, Wallingford, UK, 2009).

[35] Brito JA, Subbotin SA, Han H, Stanley JD, Dickson DW (2015). Molecular characterization of Meloidogyne christiei Golden and Kaplan, 1986 (Nematoda, Meloidogynidae) topotype population infecting Turkey oak (Quercus laevies) in Florida. J Nematol.

[36] Gerič Stare Barbara, Aydınlı Gökhan, Devran Zübeyir, Mennan Sevilhan, Strajnar Polona, Urek Gregor, Širca Saša (2019). Recognition of species belonging to Meloidogyne ethiopica group and development of a diagnostic method for its detection. European Journal of Plant Pathology.

[37] Negretti RR (2017). Characterisation of a *Meloidogyne* species complex parasitising rice in southern Brazil. Nematology.

[38] Da Silva Mattos V (2018). Integrative taxonomy of *Meloidogyne oryzae* (Nematoda: Meloidogyninae) parasitizing rice crops in Southern Brazil. Eur J Plant Pathol.

[39] Szitenberg A (2017). Comparative genomics of apomictic root-knot nematodes: hybridization, ploidy, and dynamic genome change. Genome Biol Evol.

[40] Jepson, S.B. Identification of root-knot nematode (*Meloidogyne* species) (CAB International, Wallingford, UK, 1987).

[41] Triantaphyllou AC (1985). Gametogenesis and the chromosomes of *Meloidogyne nataliei*: Not typical of other root-knot nematode. J Nematol.

[42] Wan Y (2013). A phylogenetic analysis of the grape genus (*Vitis* L.) reveals broad reticulation and concurrent diversification during Neogene and Quaternary climate change. BMC Evol Biol.

[43] Goldstein P, Triantaphyllou AC (1986). The synaptonemal complex of *Meloidogyne nataliei* and its relationship to that of other *Meloidogyne* species. Chromosoma.

[44] Phani V, Bishnoi S, Sharma A, Davies KG, Rao U (2018). Characterization of *Meloidogyne indica* (Nematoda: Meloidogynidae) parasitizing neem in India, with a molecular phylogeny of the species. J Nematol.

[45] Triantaphyllou, A. C. Cytogenetics, cytotaxonomy and phylogeny of root-knot nematodes in *An Advanced Treatise on Meloidogyne*. *Vol I*, *Biology and* Contro*l*. (eds Sasser, J. N. & Carter, C. C.) 113–126 (North Carolina State University Graphics, Raleigh, NC, USA, 1985).

[46] Triantaphyllou AC (1987). Cytogenetic status of *Meloidogyne* (*Hypsoperine*) *spartinae* in relation to other *Meloidogyne* species. J Nematol.

[47] Ryss, A.Y. *World fauna of the root parasitic nematodes of the family Pratylenchidae (Tylenchida)*. (Leningrad, USSR, Nauka, 1988).

[48] Subbotin SA, Sturhan D, Chizhov VN, Vovlas N, Baldwin JG (2006). Phylogenetic analysis of Tylenchida Thorne, 1949 as inferred from D2 and D3 expansion fragments of the 28S rRNA gene sequences. Nematology.

[49] Castagnone-Sereno P, Danchin EG, Perfus-Barbeoch L, Abad P (2013). Diversity and evolution of root-knot nematodes, genus *Meloidogyne*: new insights from the genomic era. Annu Rev Phytopathol.

[50] Castagnone-Sereno P (2006). Genetic variability and adaptive evolution in parthenogenetic root-knot nematodes. Heredity.

[51] Barker, K.R. Nematode extraction and bioassays in *An advanced treatise on Meloidogyne*, *Volume II*. *Methodology* (eds Barker, K. R., Carter, C. C. & Sasser, J. N.) 19–35 (North Carolina State University, Raleigh, NC, USA, 1985).

[52] Brito JA, Powers TO, Mullin PG, Inserra RN, Dickson DW (2004). Morphological and molecular characterization of *Meloidogyne mayaguensis* isolates from Florida. J Nematol.

[53] Esbenshade PR, Triantaphyllou AC (1985). Use of enzyme phenotypes for identification of *Meloidogyne* species. J Nematol.

[54] Tanha Maafi Z, Subbotin SA, Moens M (2003). Molecular identification of cyst-forming nematodes (Heteroderidae) from Iran and a phylogeny based on the ITS sequences of rDNA. Nematology.

[55] Subbotin SA, Waeyenberge L, Moens M (2000). Identification of cyst forming nematodes of the genus *Heterodera* (Nematoda: Heteroderidae) based on the ribosomal DNA-RFLPs. Nematology.

[56] Chizhov VN, Chumakova OA, Subbotin SA, Baldwin JG (2006). Morphological and molecular characterization of foliar nematodes of the genus *Aphelenchoides: A. fragariae and A. ritzemabosi* (Nematoda: Aphelenchoididae) from the Main Botanical Garden of the Russian Academy of Sciences, Moscow. Russ J Nematol.

[57] Derycke S, Vanaverbeke J, Rigaux A, Backeljau T, Moens T (2010). Exploring the use of cytochrome oxidase c subunit 1 (COI) for DNA barcoding of free-living marine nematodes. PLoS ONE.

[58] Powers TO, Mullin PG, Harris TS, Sutton LA, Higgins RS (2005). Incorporating molecular identification of *Meloidogyne* spp. into a large-scale regional nematode survey. J Nematol.

[59] Castresana J (2000). Selection of conserved blocks from multiple alignments for their use in phylogenetic analysis. Mol Biol Evol.

[60] Miller, M., Pfeiffer, W. & Schwartz, T. Creating the CIPRES science gateway for inference of large phylogenetic trees. In *Proceedings of the Gateway Computing Environments Workshop (GCE)* 1–8 (USA, 2010).

[61] Ronquist F (2012). MrBayes 3.2: efficient Bayesian phylogenetic inference and model choice across a large model space. Syst Biol.

[62] Stamatakis Alexandros (2014). RAxML version 8: a tool for phylogenetic analysis and post-analysis of large phylogenies. Bioinformatics.

[63] Darriba D, Taboada GL, Doallo R, Posada D (2012). jModelTest 2: more models, new heuristics and parallel computing. Nat Methods.

[64] Bandelt H, Forster P, Röhl A (1999). Median-joining networks for inferring intraspecific phylogenies. Mol Biol Evol.

