# Supplementary material for: Multigene phylogeny of root-knot nematodes and molecular characterization of Meloidogyne nataliei Golden, Rose & Bird, 1981 (Nematoda: Tylenchida)
Source: Sci Rep. 2019 Aug 13;9:11788. doi: 10.1038/s41598-019-48195-0 (PMC6692364; doi:10.1038/s41598-019-48195-0)
Supplement: Supplementary file 1 — Supplementary information [file 41598_2019_48195_MOESM1_ESM.pdf]

Multigene phylogeny of root-knot nematodes and molecular characterization of *Meloidogyne nataliei* Golden, Rose & Bird, 1981 (Nematoda: Tylenchida)

Sergio Álvarez-Ortega, Janete A. Brito & Sergei. A. Subbotin

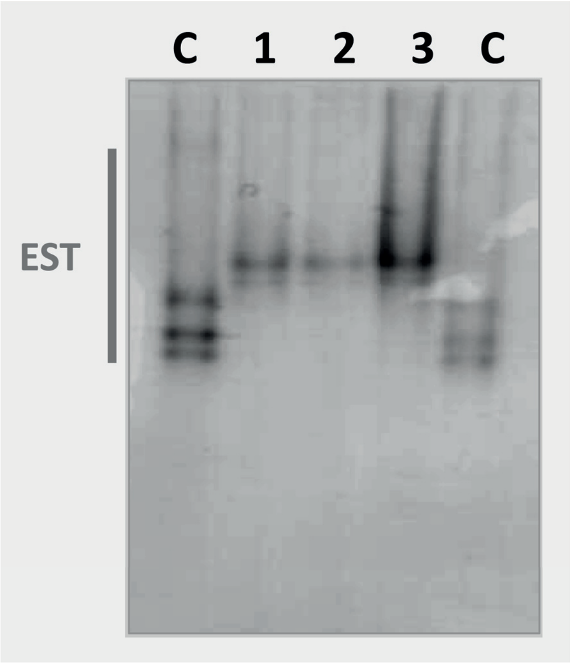

Figure S1. Esterase phenotypes of *M. natalie* and *M. javanica* (control). Lanes: 1 and 2 - extract from six females of *M. nataliei* (S1) per well; 3 - extract from ten females of *M. nataliei* (S1) per well; C - extract from one female of *M. javanica* (J3) per well.

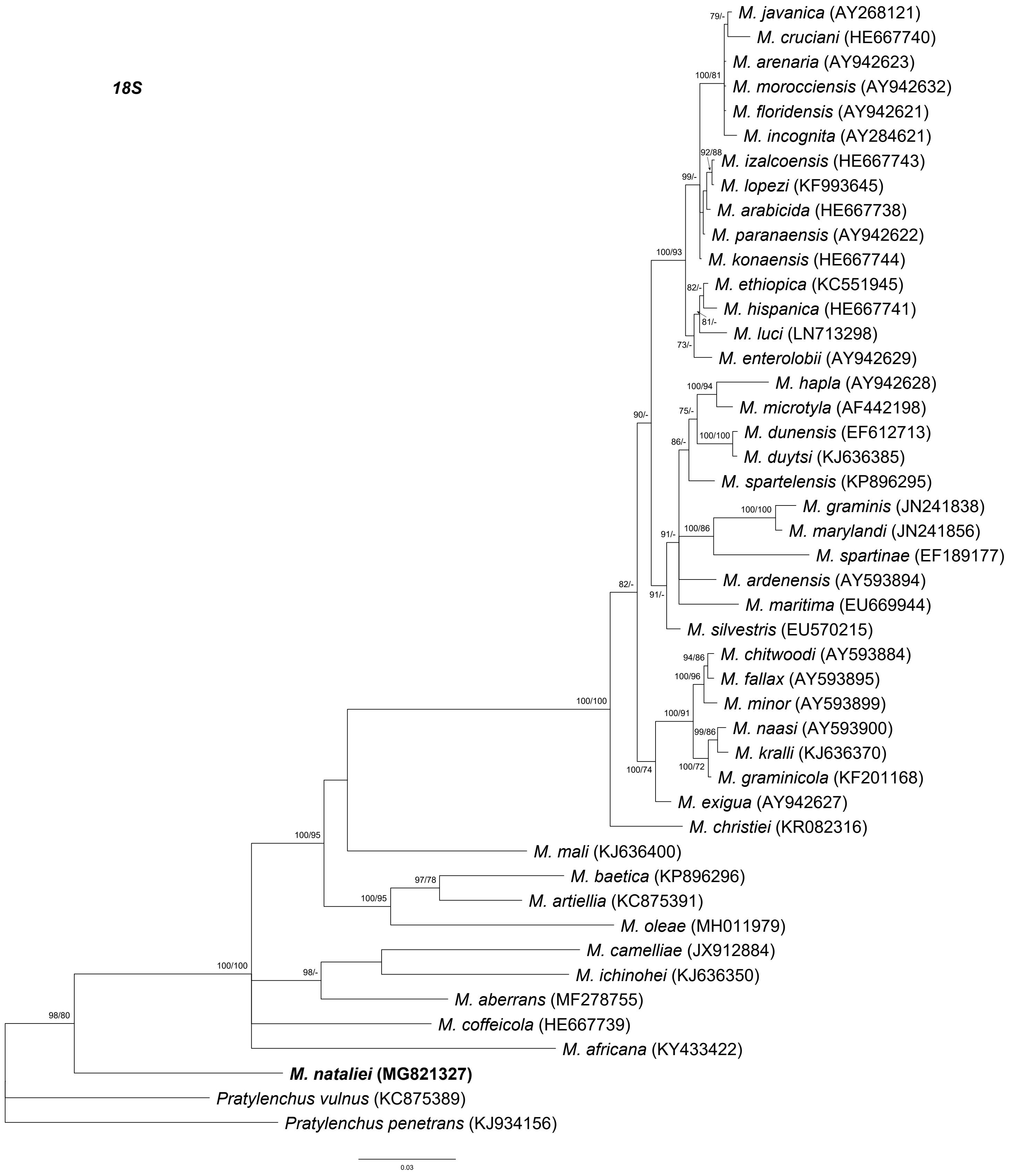

Figure S2. Bayesian 50% majority rule consensus tree as inferred from 18S rRNA gene sequence alignments under the GTR + I + G model. Branch support of over 70% is given for appropriate clades and it is indicated as: posterior probabilities value in Bayesian inference analysis/bootstrap value from maximum-likelihood analysis.

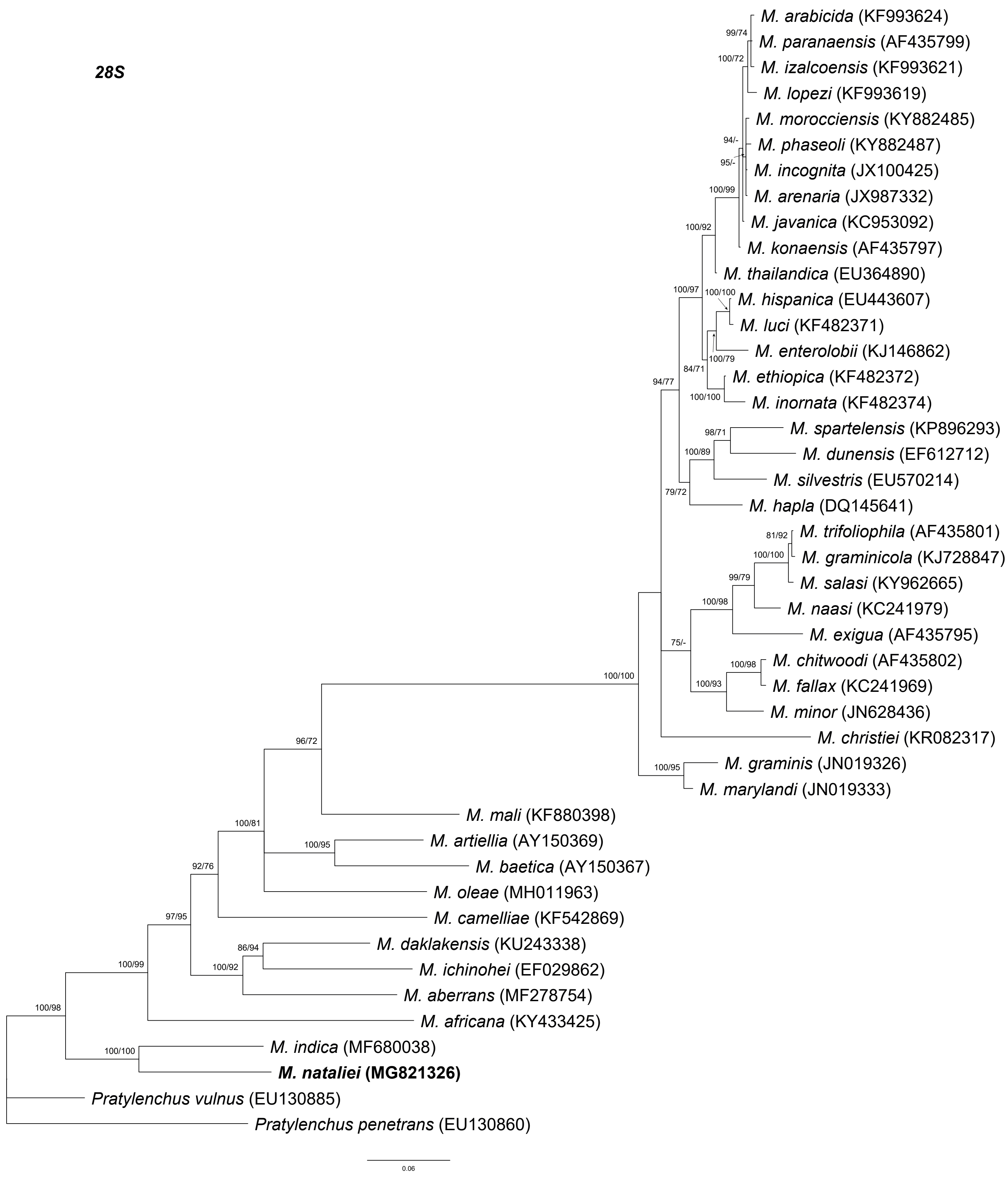

Figure S3. Bayesian 50% majority rule consensus tree as inferred from D2-D3 expansion segments of 28S rRNA gene sequence alignments under the GTR + I + G model. Branch support of over 70% is given for appropriate clades and it is indicated as: posterior probabilities value in Bayesian inference analysis/bootstrap value from maximum-likelihood analysis.

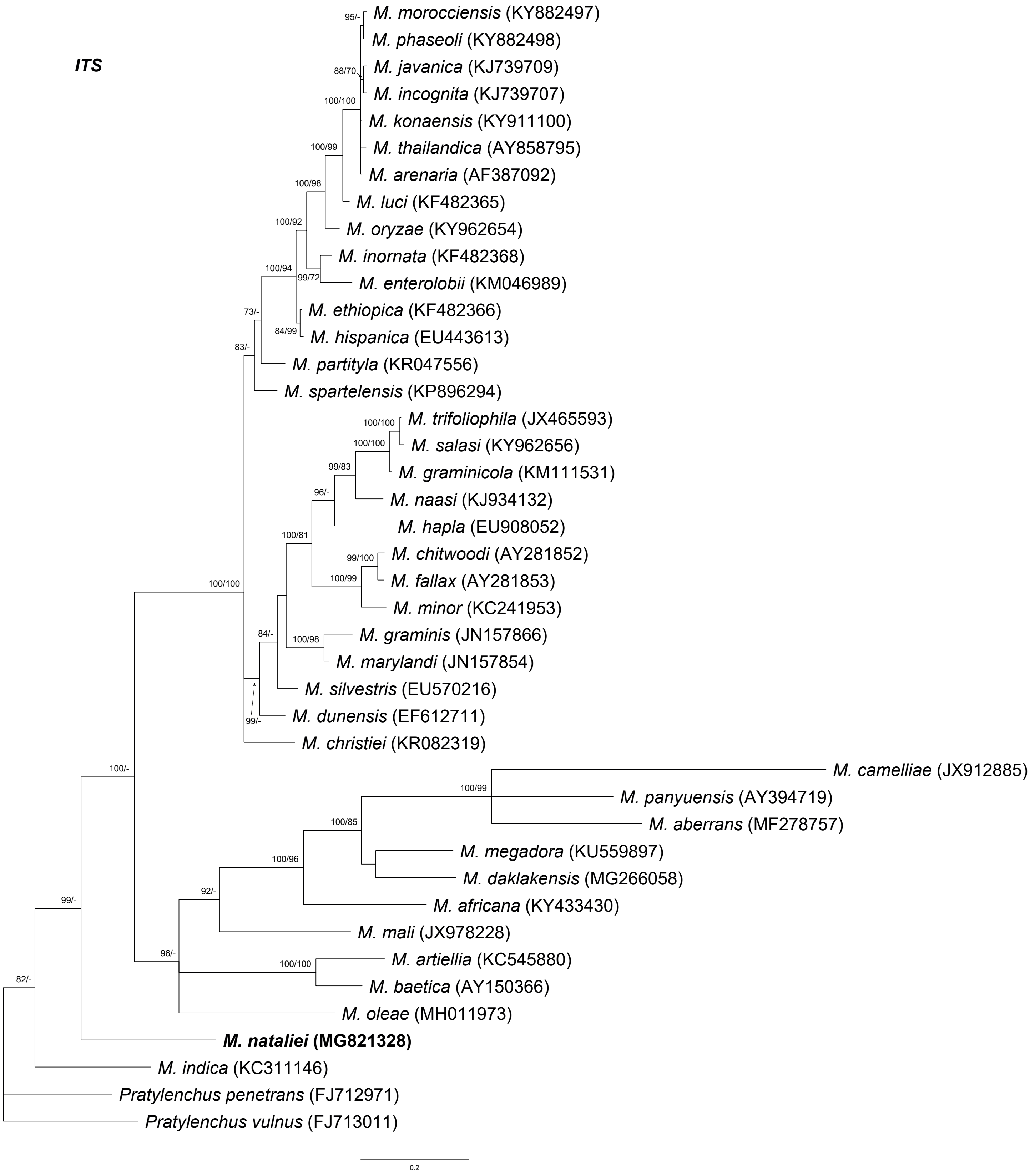

Figure S4. Bayesian 50% majority rule consensus tree as inferred from ITS1 rRNA gene sequence alignments under the GTR + G model. Branch support of over 70% is given for appropriate clades and it is indicated as: posterior probabilities value in Bayesian inference analysis/bootstrap value from maximum-likelihood analysis.

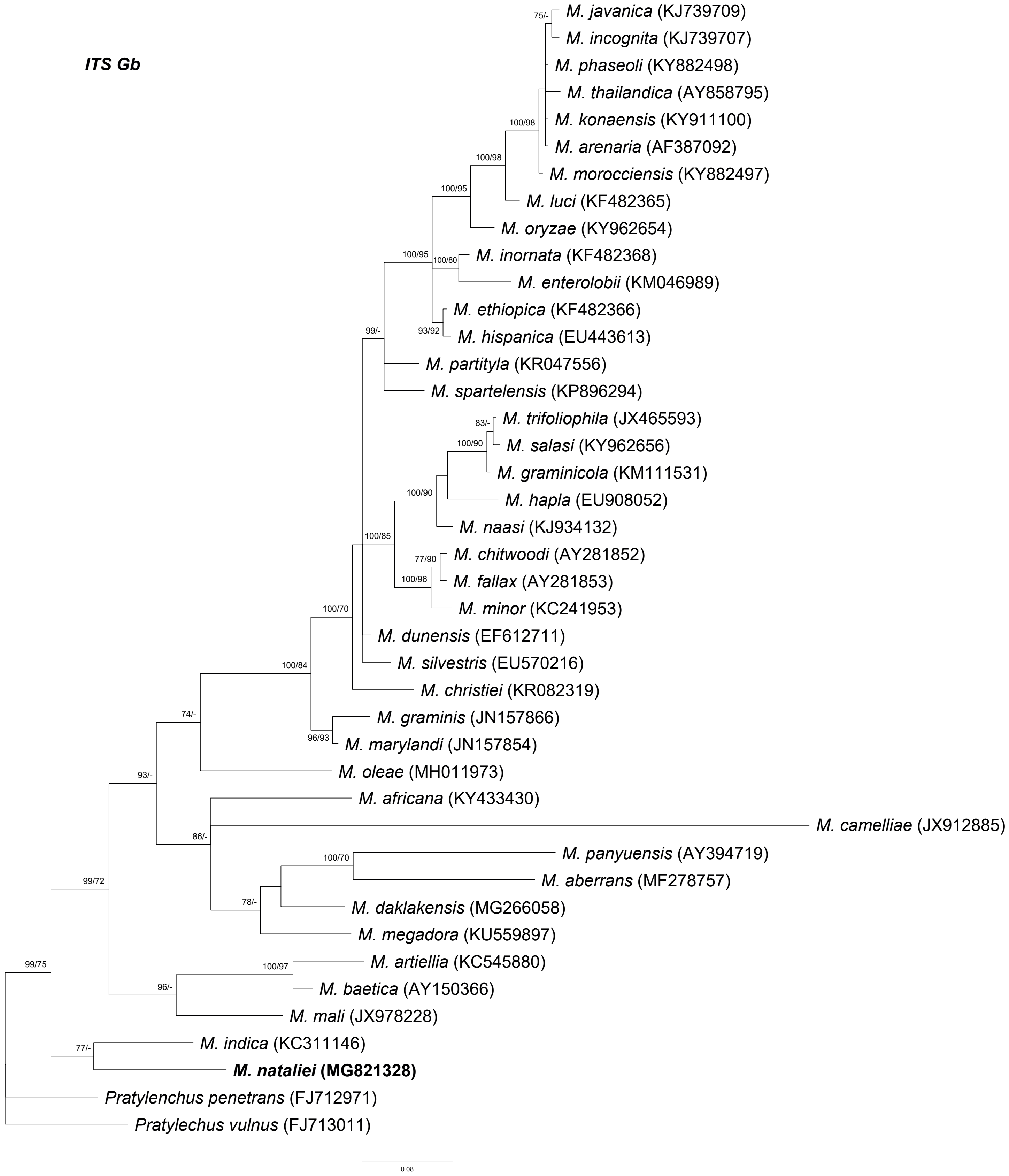

Figure S5. Bayesian 50% majority rule consensus tree as inferred from ITS1 rRNA gene sequence alignments deleting the divergent regions, under the GTR + G model. Branch support of over 70% is given for appropriate clades and it is indicated as: posterior probabilities value in Bayesian inference analysis/bootstrap value from maximum-likelihood analysis.

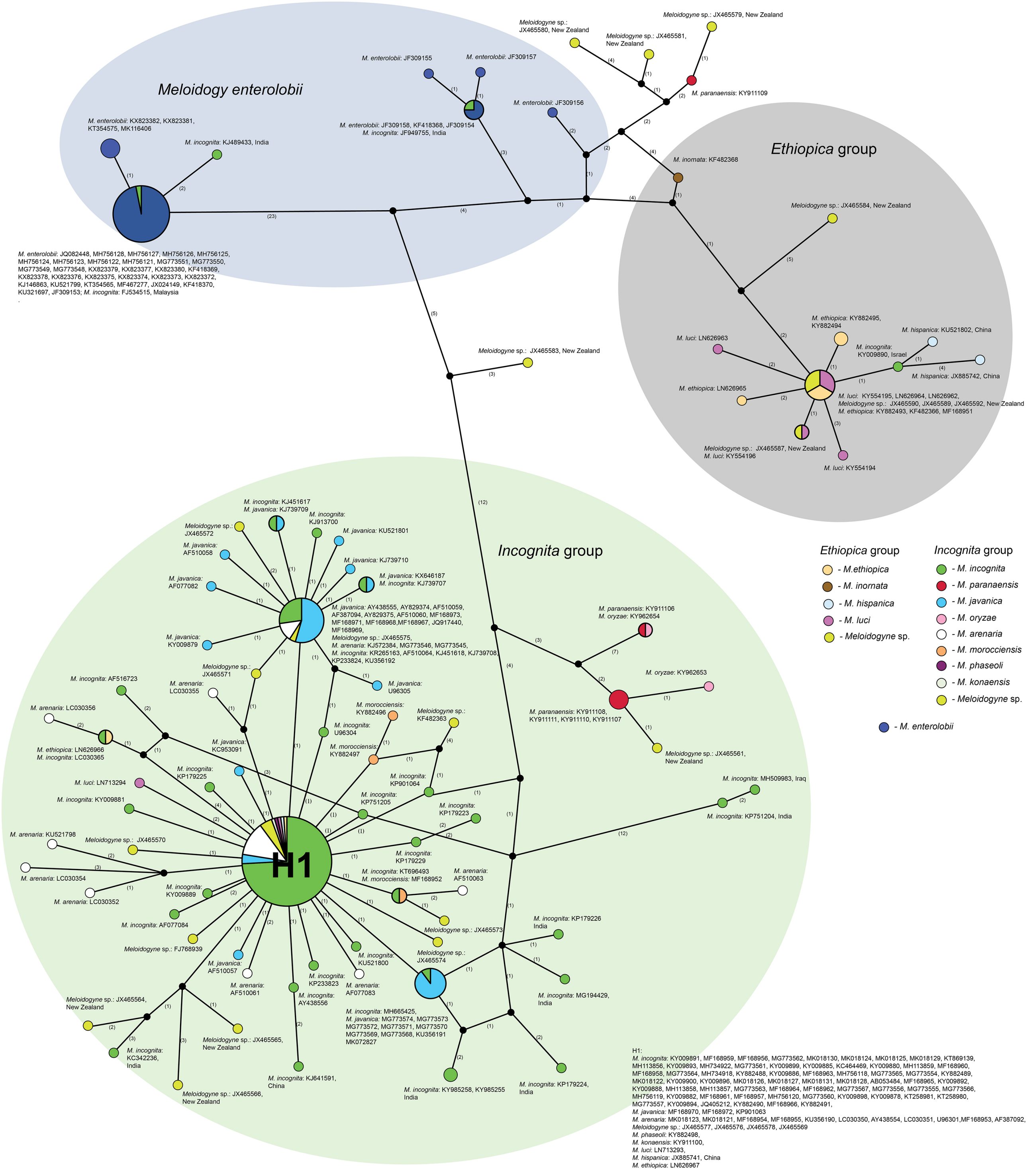

Figure S6. Statistical parsimony network showing the phylogenetic relationships between ITS rRNA gene haplotypes of species from the clade I. The sequences of each species are marked by different colors. Pies (circles) represent sequences of each species with the same haplotype and their size is proportional to the number of these sequences in the samples. Numbers of nucleotide differences between the sequences are indicated on lines connecting the pies. Small black circles represent missing haplotypes.

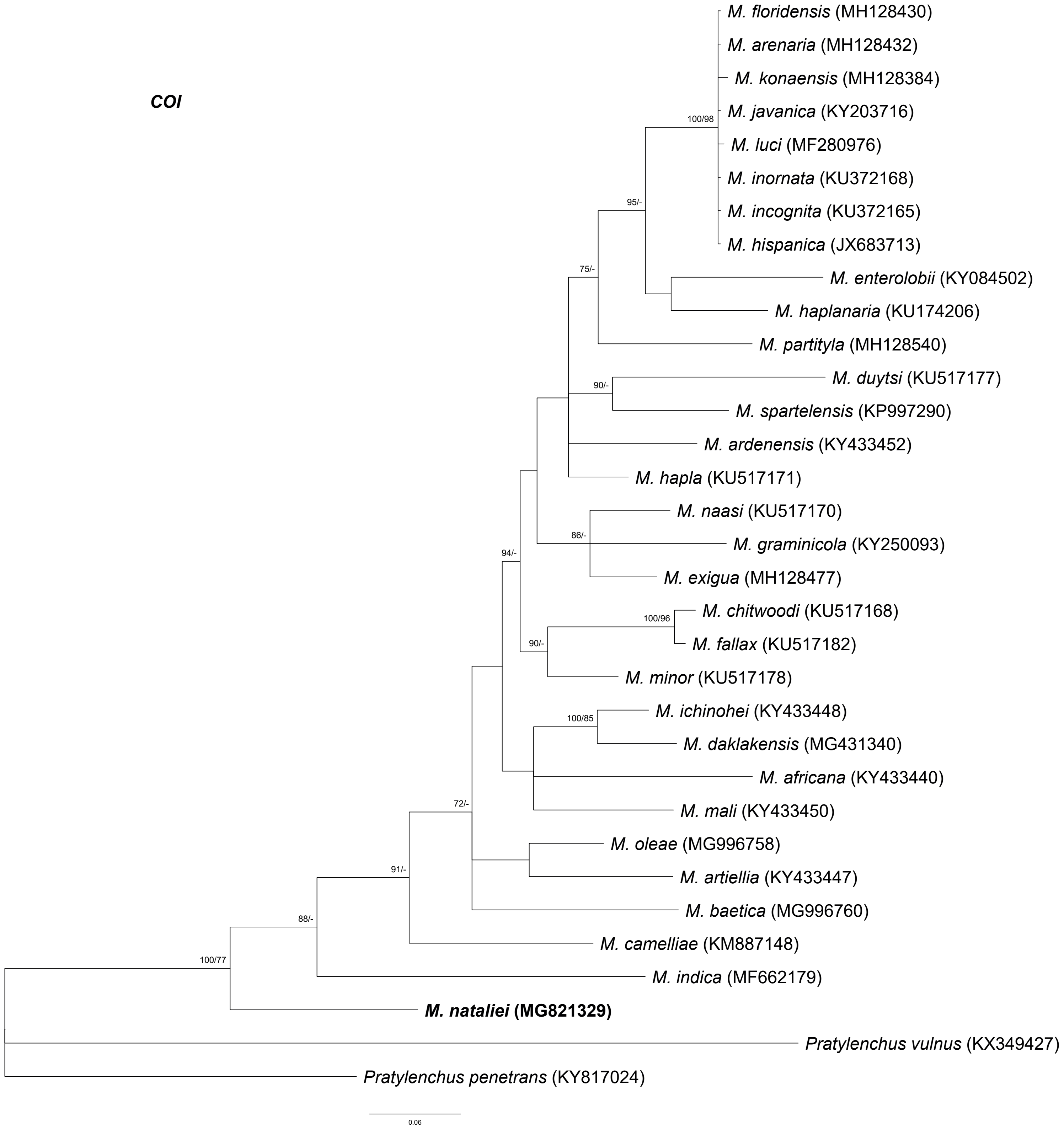

Figure S7. Bayesian 50% majority rule consensus tree as inferred from *COI* gene sequence alignments under the GTR + I + G model. Branch support of over 70% is given for appropriate clades and it is indicated as: posterior probabilities value in Bayesian inference analysis/bootstrap value from maximum-likelihood analysis.

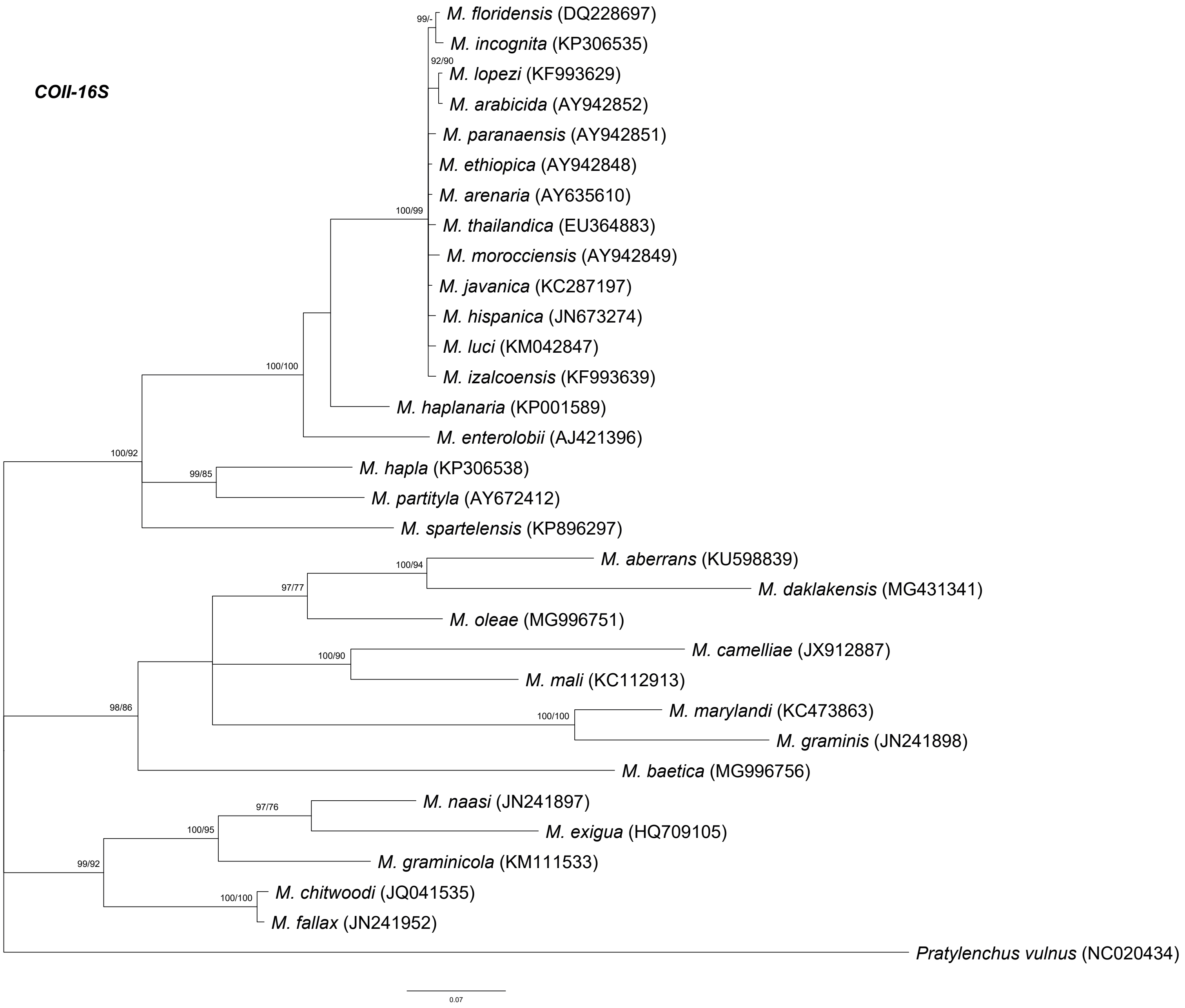

Figure S8. Bayesian 50% majority rule consensus tree as inferred from *COII-16S rRNA* gene sequence alignments under the GTR + I + G model. Branch support of over 70% is given for appropriate clades and it is indicated as: posterior probabilities value in Bayesian inference analysis/bootstrap value from maximum-likelihood analysis.

Table S1. GenBank accession numbers for gene sequences of *Meloidogyne* species used in the present phylogenetic analysis.

Species	Gene fragment				
	18S rRNA	D2-D3 of 28S rRNA	ITS rRNA	*COI*	*COII*-16S rRNA
M. abberans	MF278755	MF278754	MF278757	-	KU598839
M. africana	KY433422	KY433425	KY433430	KY433440	-
M. arabicida	HE667738	KF993624	-	-	AY942852
M. ardenensis	AY593894	-	-	KY433452	-
M. arenaria	AY942623	JX987332	AF387092	MH128432	AY635610
M. artiellia	KC875391	AY150369	KC545880	KY433447	-
M. baetica	KP896296	AY150367	AY150366	MG996760	MG996756
M. camelliae	JX912884	KF542869	JX912885	KM887148	JX912887
M. chitwoodi	AY593884	AF435802	AY281852	KU517168	JQ041535
M. christiei	KR082316	KR082317	KR082319	-	-
M. coffeicola	HE667739	-	-	-	-
M. cruciani	HE667740	-	-	-	-
M. daklakensis	-	KU243338	MG266058	MG431340	MG431341
M. dunensis	EF612713	EF612712	EF612711	-	-
M. duytsi	KJ636385	-	-	KU517177	-
M. enterolobii	AY942629	KJ146862	KM046989	KY084502	AJ421396
M. ethiopica	KC551945	KF482372	KF482366	-	AY942848
M. exigua	AY942627	AF435795	-	MH128477	HQ709105
M. fallax	AY593895	KC241969	AY281853	KU517182	JN241952
M. floridensis	AY942621	-	-	MH128430	DQ228697
M. graminicola	KF201168	KJ728847	KM111531	KY250093	KM111533
M. graminis	JN241838	JN019326	JN157866	-	JN241898
M. hapla	AY942628	DQ145641	EU908052	KU517171	KP306538
M. haplanaria	-	-	-	KU174206	KP001589
M. hispanica	HE667741	EU443607	EU443613	JX683713	JN673274
M. ichinohei	KJ636350	EF029862	-	KY433448	-
M. incognita	AY284621	JX100425	KJ739707	KU372165	KP306535
M. indica		MF680038	KC311146	MF662179	-
M. inornata	-	KF482374	KF482368	KU372168	-
M. izalcoensis	HE667743	KF993621	-	-	KF993639
M. javanica	AY268121	KC953092	KJ739709	KY203716	KC287197
M. konaensis	HE667744	AF435797	KY911100	MH128384	-
M. kralli	KJ636370	-	-	-	-
M. lopezi	KF993645	KF993619	-	-	KF993629
M. luci	LN713298	KF482371	KF482365	MF280976	KM042847
M. mali	KJ636400	KF880398	JX978228	KY433450	KC112913
M. maritima	EU669944	-	-	-	-
M. marylandi	JN241856	JN019333	JN157854	-	KC473863
M. megadora	-	-	KU559897	-	-

M. microtyla	AF442198	-	-	-	-
M. minor	AY593899	JN628436	KC241953	KU517178	-
M. morocciensis	AY942632	KY882485	KY882497	-	AY942849
M. naasi	AY593900	KC241979	KJ934132	KU517170	JN241897
M. nataliei	**MG821327**	**MG821326**	**MG821328**	**MG821329**	-
M. oleae	MH011979	MH011963	MH011973	MG996758	MG996751
M. oryzae	-	-	KY962654	-	-
M. panyuensis	-	-	AY394719	-	-
M. paranaensis	AY942622	AF435799	-	-	AY942851
M. partityla	-	-	KR047556	MH128540	AY672412
M. phaseoli	-	KY882487	KY882498	-	-
M. salasi	-	KY962665	KY962656	-	-
M. silvestris	EU570215	EU570214	EU570216	-	-
M. spartelensis	KP896295	KP896293	KP896294	KP997290	KP896297
M. spartinae	EF189177	-	-	-	-
M. thailandica	-	EU364890	AY858795	-	EU364883
M. trifoliophila	-	AF435801	JX465593	-	-
P. penetrans	KJ934156	EU130860	FJ712971	KY817024	-
P. vulnus	KC875389	EU130885	FJ713011	KX349427	NC020434
